# Determination of Carbohydrate Composition in Lentils Using Near-Infrared Spectroscopy

**DOI:** 10.3390/s24134232

**Published:** 2024-06-29

**Authors:** Rocío López-Calabozo, Ângela Liberal, Ângela Fernandes, Isabel Revilla, Isabel C. F. R. Ferreira, Lillian Barros, Ana M. Vivar-Quintana

**Affiliations:** 1 Food Technology Area, Escuela Politécnica Superior de Zamora, Universidad de Salamanca, Avenida Requejo, 33, 49022 Zamora, Spain; rociolc2112@gmail.com (R.L.-C.); angela__lib@hotmail.com (Â.L.); irevilla@usal.es (I.R.); 2 Centro de Investigaçao de Montanha (CIMO), Instituto Politécnico de Bragança, Campus de Santa Apolónia, 5300-253 Bragança, Portugal; afeitor@ipb.pt (Â.F.); iferreira@ipb.pt (I.C.F.R.F.); lillian@ipb.pt (L.B.); 3 Laboratório para a Sustentabilidade e Tecnologia em Regiões de Montanha, Instituto Politécnico de Bragança, Campus de Santa Apolónia, 5300-253 Bragança, Portugal

**Keywords:** lentil, carbohydrates, NIR, fibre, starch, sugars

## Abstract

Carbohydrates are the main components of lentils, accounting for more than 60% of their composition. Their content is influenced by genetic factors, with different contents depending on the variety. These compounds have not only been linked to interesting health benefits, but they also have a significant influence on the techno-functional properties of lentil-derived products. In this study, the use of near-infrared spectroscopy (NIRS) to predict the concentration of total carbohydrate, fibre, starch, total sugars, fructose, sucrose and raffinose was investigated. For this purpose, six different cultivars of macrosperm (n = 37) and microsperm (n = 43) lentils have been analysed, the samples were recorded whole and ground and the suitability of both recording methods were compared. Different spectral and mathematical pre-treatments were evaluated before developing the calibration models using the Modified Partial Least Squares regression method, with a cross-validation and an external validation. The predictive models developed show excellent coefficients of determination (RSQ > 0.9) for the total sugars and fructose, sucrose, and raffinose. The recording of ground samples allowed for obtaining better models for the calibration of starch content (R > 0.8), total sugars and sucrose (R > 0.93), and raffinose (R > 0.91). The results obtained confirm that there is sufficient information in the NIRS spectral region for the development of predictive models for the quantification of the carbohydrate content in lentils.

## 1. Introduction

Lentils (*Lens culinaris* Med.) are considered a basic food in the Mediterranean diet and are an important source of low-cost protein in developing countries [1]. They play an important role in the development of sustainable agriculture because their cultivation incorporates nitrogen, carbon, and organic matter into soils [2]. In recent years, the consumption of lentils has increased around the world due to their incorporation into healthy and sustainable diets, with environmental, economic, health and nutritional reasons are behind this increase [3].

The nutritional composition of lentil seeds is characterised by high protein and low-fat contents, as well as being rich in minerals and vitamins. Although much attention has been focused on the high protein content of lentils, carbohydrates are the major compound in lentil seeds (62–69%), consisting mainly of starch (35–53%) [4] and fibre (5–20%) [5]. Besides these, other carbohydrates have also been described in lentils as oligosaccharides of the raffinose family (4.14%) with stachyose being the major compound. In addition, lentils contain simple sugars (1.75%), mainly sucrose, and sugar alcohols (0.7%), primarily sorbitol [6]. Many of the health benefits attributed to lentils have been linked to their type and concentration of carbohydrates [7,8]. Lentils have a low glycaemic index, which has been linked to their beneficial effect on some diseases [9] with regular consumption being associated with a lower risk of developing type 2 diabetes, coronary heart disease, and some forms of cancer [10]. In addition, its consumption has been associated with a lower rate of obesity and metabolic syndrome [11]. Consumption of pulses has been shown to have beneficial effects by consistently reducing acute blood glucose and insulin response compared to other starchy control foods [12]. In addition, lentil carbohydrates have attractive functional properties which are particularly interesting in the case of lentil flours. Thus, the lentil starch offers a higher gelling property than starch from cereals or tubers [13].

Significant differences in carbohydrate concentration have been found in lentils grown in different countries. The type and concentration of carbohydrates present in lentils have been related to the soil and climatic conditions, and methods of cultivation as well as to the genotype [8]. Thus, Johnson et al. [14] reported that total concentrations of low molecular weight carbohydrates were generally higher in regions with lower rainfall, higher temperatures, and higher estimated stress index. Plaza et al. [15] found that the harvest year affects the concentration of total carbohydrates and fibre in lentils. Variety also has an influence, with green lentils showing higher concentrations of carbohydrates than red lentils [16]. In addition, different lentil genotypes seem to differ in their enzyme composition which influences the accumulation of soluble carbohydrates in the seed [14].

The possibility to carry out rapid quantification of the carbohydrate content of lentils can benefit lentil breeding programmes to select seeds with higher carbohydrate content. It can also help their application as an ingredient or fortifier in the development of new products. However, conventional reference methods for the determination of these compounds are laborious, expensive and time-consuming. In this context near- infrared spectroscopy (NIRS) is a non-destructive, powerful, and fast tool that is increasingly being used for food quality assessment in recent years [17]. NIR spectroscopy in combination with chemometric analysis has been used in the study of non-structural carbohydrates (NSCs) in plants [18]. Moreover, this methodology has been applied to a wide variety of foods for the prediction of their carbohydrate composition, such as maize, sweet potato, apple, mango, apricot, onion, rice, or sorghum. Thus, the prediction of monosaccharides (glucose and fructose) and disaccharides (sucrose) [19,20,21,22], polysaccharides (fructans) [23], cellulose, lignin and hemicellulose [24], starch [25,26,27,28], and amylose [29] content was carried out. 

Regarding its application in lentils, NIRS has been used to determine protein concentration [30], colour [31], moisture, fibre, ash and total fat, quantification of fatty acids and mineral composition [32] as well as in the prediction of the protein and amino acid content [33]. It has also been applied to the evaluation of the nutritional quality of green and red lentils [34] and in recent studies, the feasibility of its application for the geographic origin discrimination of lentils has been evaluated [5,35]. However, to our knowledge, NIR technology has not been applied to assess carbohydrate composition in lentils. In recent years, there has been a significant increase in lentil consumption that has been linked to its health benefits, especially the reduction of the risk of diseases such as obesity, diabetes or hypertension [4]. At the same time, the use of lentil-derived products, such as lentil flour, has also increased. Bakery products and extruded products have been developed from this flour, and it has been used for the partial substitution of other ingredients in the preparation of dairy and meat products. Considering the health implications and functional properties associated with lentil carbohydrates, the aim of this work is to study the feasibility of NIRS technology for the quantitative determination of fibre, starch, total carbohydrates, and free sugar composition in lentils. It is of great importance for the food industry to have a tool that would allow rapid estimation of its carbohydrate composition.

## 2. Materials and Methods

### 2.1. Samples

A total of 80 lentil samples have been analysed. These samples belong to six different cultivars: four microsperm and two macrosperm (Table A1). Of all the samples, 500 g of lentils were available. Some samples were supplied directly by the Legume Center (Salamanca) and others were purchased in specialised pulse shops. 

### 2.2. Chemical Composition

The composition of lentils was evaluated according to AOAC procedures [36]. The moisture content was determined using the AACC method 14–15 A. The macro-Kjeldahl method (AOAC 950.36) was used to determine the crude protein content (N × 6.25), and the ash content (AACC method 08-01.01) was determined by incineration of the samples at 550 ± 10 °C.

Starch content was determined according to the methods described by Alajaji and El-Adawy [37] as reducing sugars after complete acid hydrolysis. The total amount of carbohydrates was assessed using the difference ((100 − (Ash + Protein + Total Fat)). Dietary fibres were determined according to the enzymatic–gravimetric method by enzymatic digestion with α-amylase, protease, and amyloglucosidase. Samples were analysed in duplicate, and results were expressed as relative percentages according to the equation: % total dietary fibre = [(R − P − A)/SW] × 100, where R is the average residue, P is the average protein, A is the average ash and SW is the average weight of the samples. Subsequently, the protein and ash content of the digested residues was analysed. Reducing sugars were analysed by High-Performance Liquid Chromatography coupled to a refractive index refraction detector (HPLC-RI) according to the method described by Liberal et al. [38]. Melezitose was used as an internal standard. Concentrations of free sugars were expressed in g per 100 g of fresh weight (fw) based on calibration equations using fructose, sucrose, glucose, trehalose, and raffinose standards from Sigma–Aldrich (St. Louis, MO, USA).

### 2.3. NIR Spectroscopy

Lentil spectra were recorded with a Foss NIRSystem 5000 (Foss Iberia, Barcelona, Spain) equipped with a fibre optic probe of remote reflectance, type 210/210 of 1.5 m (Ref no. R6539-A). The probe has a quartz window of 5 cm × 5 cm and the reflectance is measured in the wavelength region between 1100 and 2000 nm. The spectra were recorded each 2 nm, performing 32 scans of the sample and of the reference. To minimise the error, the samples were analysed in triplicate, and the mean spectrum was accepted attending to the Mahalanobis constant criteria (H < 3). The software used was Win ISI 4.10 (Foss Iberia, S.A., Barcelona, Spain). 

The recording of the spectrum was carried out on whole lentils and on ground lentils, comparing the results obtained in both cases. The records of the spectra were obtained by direct application of the probe over 10 g of sample. To obtain the ground samples, the process described by Liberal et al. [38] was followed. Fifty grams of each sample with the skin, were ground in a Foss Knifetec™ 1095 mill (Foss Iberia, Barcelona, Spain) with the temperature controlled at 20 °C. To obtain a uniform particle size, samples were graded on a 60-mesh sieve. 

### 2.4. PCA Analysis and NIR-Chemometric Methods

The lentil samples were divided into two groups: 80% (64 samples) were used for the development of a calibration model, and the remaining 20% (16 samples) were used for further external validation of the developed calibration models. The distribution of the samples in these two groups was randomised. Both sets were evaluated to ensure that they were similar in the concentration of the compounds to be calibrated (Table A2).

Different spectral and mathematical pre-treatments were applied to the spectra, evaluating which offered the best results in the development of the predictive models. Four spectral pre-treatments: multiplicative scattering correction (MSC), Standard Normal Variate (SNV), DeTrend (DT), and the combined method (SNV-DT) were evaluated. In addition, as the derivative applied in the mathematical pre-treatment has a great influence on the predictive ability of the model [39], five mathematical pre-treatments (0, 0, 1, 1; 1, 4, 4, 1; 1, 10, 10, 1; 2, 4, 4, 1; 2, 10, 10, 1) were evaluated. The first digit (derivatives) indicates the derivative applied and the second digit (gap in derivatives) indicates the number of points over which the derivative was applied. The third and fourth numbers refer to different spectrum smoothing.

The different carbohydrates analysed together with the spectral data obtained from the NIR recording of the samples were used for the development of calibration models using the Modified Partial Least Squares (MPLS) method. The statistical parameters of the calibration were obtained for each of the analytical parameters after removing the samples for spectral (H-criterion) or chemical (T-criterion) reasons [32]. The equations developed through multivariate calibration are based on a set of absorption values of several wavelengths. The correlation between the concentration of the analysed parameters and their spectral absorption at different wavelengths is given by the expression: *y* = *β*0 + *β*1 *X*1 + *β*2 *X*2 + *β*3 *X*3 + … + *β*n *Xv* where *β* are the coefficients and *X*1, *X*2, *X*3, … *Xn* are the wavelengths at which the correlation of the concentration of the components is maximum (in + or − value).

An internal validation was carried out with the best equation obtained for each parameter, with the same samples used for the development of the model. Subsequently, an external validation was carried out using 16 samples that had not been included in the development of the model. The results predicted by applying the developed equation and those obtained in the laboratory for the same samples were compared to establish the suitability of the model. The developed models were evaluated by analysing the Ratio Performance Deviation (RPD), the mean square error of calibration (SEC) and the standard error of prediction (SEP). The RPD is defined as the ratio between the standard deviation of the data obtained with the reference methods and the SECV. Models were considered adequate when their RPD values were greater than 2, considering that the models can be applied for approximate prediction when the RPD ranged between 2.0 and 2.5, and were considered excellent or good when the RPD was greater than 2.5. The best calibration equation was chosen based on the Multiple Correlation Coefficient (RSQ) of determination and the root mean square error of calibration (RMSEC) [5].

### 2.5. Statistical Analyses of Reference Parameters

The data were analysed using IBM SPSS Statistics (version 27) (IBM Corp., Armonk, NY, USA). Variations between samples were evaluated using a one-way analysis of variance, followed by Tukey’s significant difference post hoc test (*p* < 0.05). In the validation of the developed model, a Student *t*-test was applied considering differences between the predicted data and the reference values at *p* < 0.05.

## 3. Results

### 3.1. Carbohydrate Composition of Lentils

The composition of the different lentil varieties analysed in this study is shown in Table 1. The total carbohydrate content of the samples ranged from 64.87 g/100 g for the Pardina variety to 76.50 g/100 g for the Stone variety. Starch is the most abundant carbohydrate in all lentil varieties, while fibre content showed greater variability between varieties. The Guareña and Pardina varieties have the same average concentration of total carbohydrates, fibre, and starch. On the other hand, the varieties Castellana, Crimson, and Stone have the highest concentration of total carbohydrates and the lowest concentration of fibre. The Crimson variety has the lowest fibre content and the highest starch content. This is the only dehulled lentil variety in the study, which justifies this composition, as fibre is mainly found in the seed coat [40].

The concentration of total sugars ranged from 1.23 g/100 g (var. Pardina) to 3.40 g/100 g (var. Stone). In terms of individual sugars, sucrose is the main sugar except in the Castellana and Stone varieties being fructose in higher concentrations. Fructose was found in all the varieties analysed except the Pardina variety. The highest concentrations of raffinose were found in the Guareña and Pardina varieties, this sugar not being present in the Beluga and Stone varieties. In terms of total sugars, the microsperm varieties have the highest concentration, with fructose and sucrose being the main sugars. However, the Pardina variety has the lowest concentration of sugars. All the lentils analysed showed close moisture values, with no significant differences in this parameter except between the Beluga and Guareña varieties.

Although several studies have characterised the nutritional composition of lentils, the results are not always comparable due to the analysis methodologies employed, as well as the fact that many studies do not identify the lentil varieties analysed. Despite these difficulties, it can be considered that the total carbohydrate content of the varieties analysed is within the ranges described by other authors [8,41,42]. This content varies depending on the variety, but also on environmental factors such as climate, geographical location, harvest year, and cultivation methods [14,16,43]. In addition, starch and fibre contents are in the ranges described in the literature [42]. In the particular case of red lentils, Kaale et al. [44] also describe low fibre contents (10.78%), although the study does not indicate whether the lentils are peeled. Regarding the composition of individual sugars, Johnson et al. [8] report contents of 0.01% for fructose, 1.71% for sucrose, and 0.5% for raffinose, in agreement with those found in this study. Siva et al. [6] reported higher fructose (0.004–0.02%) and sucrose (3–10%) and similar raffinose (0.49–0.48%) contents when analysing red and green lentils. Both genotype and genotype-location combinations have been shown to have a strong influence on the concentration of low molecular-weight carbohydrates [14]. Tahir et al. [45] described the significant effects of the environment, cultivar, and their interaction on the sugar content of lentils.

Regarding the external colour of the lentils, the Crimson variety is red, the Beluga variety is black, the Stone variety is green and the Guareña, Castellana, and Pardina varieties are brown varieties. The external colour of the lentils does not show an influence on their carbohydrate composition. In terms of size, the Guareña and Castellana varieties belong to the group of macrosperma varieties, with a larger diameter, while the Pardina, Crimson, Beluga, and Stone varieties are called microsperma. In terms of size, significant differences (*p* < 0.05) were found for sucrose and raffinose content, with macrosperm lentils showing the highest concentrations. Significant differences were also observed for starch and total sugar content, with higher contents in microsperm lentils. These results are in line with those described Ramdath et al. [46] for red and green lentils, where it was found that smaller lentil seeds have lower carbohydrate contents.

Based on the above data, a large variability of carbohydrate composition has been described for lentils. Genetics combined with agro-ecological factors (temperature, soil type, rainfall), production methodology (use of pesticides, fertilisers, herbicides), and the ability of the plant to adapt to biotic and abiotic stress conditions are responsible for this variability [47]. Based on their composition, the presence of low-digestible carbohydrates, also called prebiotics, stands out. Their consumption can help to protect and maintain the health of the intestinal microbiota [48].

### 3.2. Spectral Characteristics

**Figure 1:**
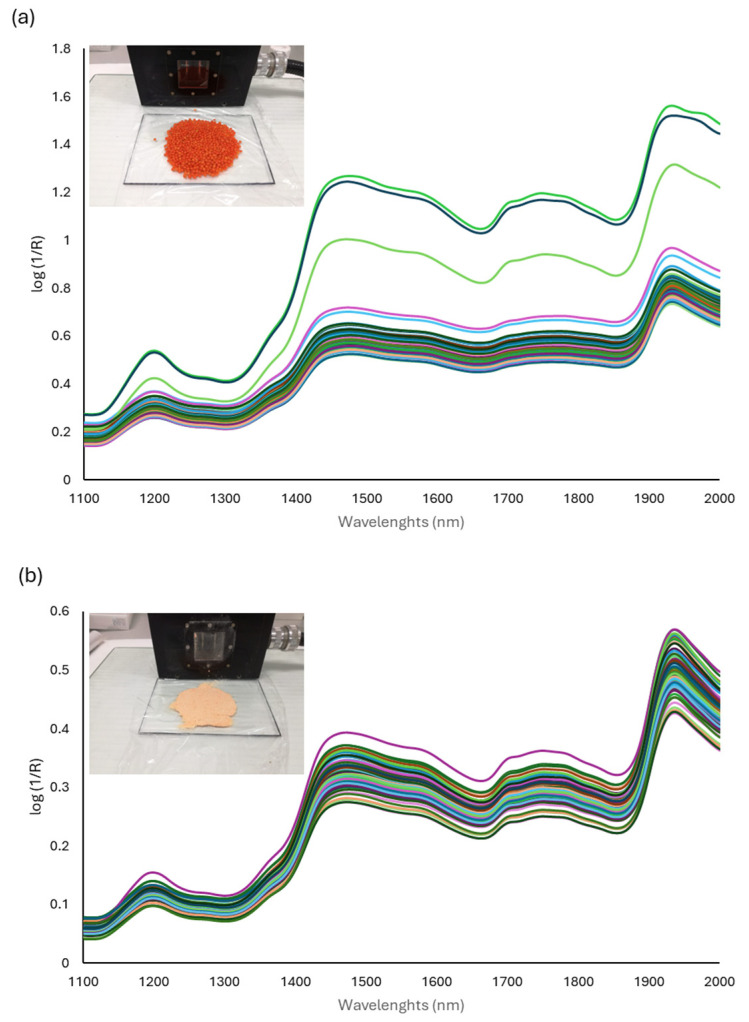
Raw spectra obtained by direct application of a remote fibre-optic probe on 10 g of sample of whole lentils (**a**) and ground lentils (**b**).

The spectra of the samples were recorded over the whole lentils (Figure 1a) and over the ground and sieved lentils (Figure 1b). Regardless of the recording method, similar absorbance zones are observed in the spectra. The spectra obtained show a higher absorbance and a greater difference in absorption intensity between samples when the lentils were recorded whole (Figure 1a). The spectra of the ground samples (Figure 1b) revealed less dispersion along the y-axis. Previous studies [32,33] have shown similar results in the spectral data of ground lentils. This is possibly due to size uniformity between samples after grinding and sieving [49]. Despite this, a small dispersion can also be observed in ground samples which Vega-Castellote et al. [50] attribute to the detector not being able to register all the light incidents on the sample.

The spectra show different absorbance bands which are combinations of molecular overtones and molecular vibrations caused by the absorbance due to different functional groups (especially -CH, -OH, and -NH). The spectra in Figure 1 show three zones of maximum absorbance between 1200–1250 nm, 1440–1500 nm and 1900–1936. Although the bands cannot be assigned to a specific compound, there are bands that have been described as characteristic of oligosaccharides and polysaccharides [51]. The absorbance maximum observed around 1200 nm corresponds to the O-H stretch first overtone. Ghosh and Roy [52] related the absorbance at 1195 nm to the glucose and the absorbance at 1202 and 1240 nm to starch in lentil samples [13]. In addition, the C-H stretching peaks due to C-H bonds of carbohydrates have been identified as the second overtone in the region 1176–1212 nm [35]. 

The increase in absorbance between 1440 and 1500 nm has been related to C-H bending of -CH2, and to the O-H stretch first overtone [13]. The absorbance at 1433 nm has been related to sucrose [53] and the 1440–1443 nm zone to crystalline sucrose [54]. In addition, peaks around 1200 nm and 1440 nm have been related to the sugar absorption bands [55]. A signal between 1900–1950 nm can also be observed in our spectra. A peak at about 1936 nm was related to a combination O-stretching/O-H stretching band of O-H bonds, particularly those of carbohydrates [35]. 

In order to determine the quality of the spectra obtained and to eliminate those with low quality, the first derivative of the original spectrum was performed. The application of the first derivative to the spectra has been proposed as a method to reveal the spectral regions where the signal-to-noise ratio is degraded [56]. The analysis of the first derivative, both in whole and ground samples, showed no noise in the spectra, so that all the spectra can be used for the subsequent calibration process.

### 3.3. Calibration Equations

Principal component analysis (PCA) to reduce the dimensionality of the data matrix while retaining the highest variability in the spectral data was performed. They were implemented on the original spectra and on the spectra after applying the pretreatments indicated in the materials and methods section. In all the PCAs performed, between 5 and 11 principal components were needed to explain a spectral variability of more than 98% in whole lentils. In the case of ground lentils, a higher number of PCs (7–14) were needed to explain the same percentage of variability. From these analyses, outlier samples were identified. Outliers were those that showed extreme spectral distances from the centre of the calibration group (H > 3) [57]. The results obtained showed that three samples were recurrently identified as outliers in the PCAs performed. These three samples were microsperm of the Crimson and Pardina varieties, which were removed for the development of the calibration models. Once the outliers were removed, the sample set was divided into two groups: a calibration set, and a validation set. The model was developed using only the samples from the calibration set as explained in the materials and methods section. Since some lentil samples had zero values for some of the parameters analysed, these were considered as values in both the calibration and validation procedures.

The statistics of the MPLS calibrations developed for whole and ground lentil samples using the NIR region (1100–2000 nm) are shown in Table 2. A total of four hundred regression equations per parameter were developed by combining five spectral derivative math treatments and four scatter correction methods. With respect to the scatter correction, the SNV or No pre-treatments showed improved results in all the parameters except for TC content in ground samples where Detrend pre-treatment was the most appropriate. Our results showed that the mathematical pre-treatment of the second derivative is the best for the calibration of carbohydrates in lentils, regardless of the parameter analysed. The most appropriate mathematical pretreatment is related to the parameter being calibrated [58] and to the food on which the analysis is performed [26,59,60].

The best of all the equations generated were chosen as those with the highest RSQ and RPD and the lowest SEC and SECV. Based on the RSQ values and attending to the classification established by Williams [61], for whole lentils, the calibration models obtained can be considered excellent (RSQ > 0.91) for fructose and sucrose, and good (RSQ > 0.82 and <0.90) for raffinose, fibre, total sugars, and total carbohydrates. However, it was not possible to develop a model that would allow an adequate prediction for starch content. For lentils that were recorded ground, excellent calibration models were obtained for total sugars, fructose, sucrose, and raffinose content. The models were good for starch and fibre while for total carbohydrates the quantitative predictions are considered approximate. 

Considering the SEC and the SECV values, these have been low in all the models developed for all the parameters analysed, being higher in those parameters where there is a higher concentration of the compound: starch, fibre, and total carbohydrates. The reason for the large difference between the SEC and SECV has not been identified, although the large presence of outliers has been suggested as one of the possible reasons. In this sense, the application of other approaches to identify and eliminate outliers would allow the reduction of these differences. According to our results, only the model for starch in whole lentils (RPD < 2) cannot be considered adequate [62]. On the other hand, the values for fibre in whole lentils and fibre, total carbohydrates, and starch in ground lentils can be applied for the approximate prediction of these parameters.

The differences in the predictive ability of the models developed depending on the method in which the spectra were recorded (whole or ground lentils) might be related to the distribution of the different compounds in lentil seed. The two most important macrostructural parts of lentils, as in all legumes, are the cotyledon and the seed coat. Regarding carbohydrates, cotyledon contains starch and some non-starch polysaccharides [63] as well as low molecular weight carbohydrates such as mono- and disaccharides and raffinose-family oligosaccharides [64]. Meanwhile, the seed coat contains mainly no starch carbohydrates (cellulose, hemicelluloses, and pectins). In the starch case, it is encapsulated in the parenchyma cells by primary cell walls within the cotyledon [65], which would explain why it is not possible to find a model to predict its concentration when samples are recorded whole. However, by grounding the lentil it would be possible to find free starch grains [66] which would facilitate the interaction of the starch with the incident light of the NIR.

Table 2 also lists the number of samples (n) used in the development of the model after eliminating the outliers. Attending to the n value, the case of fructose is remarkable where, regardless of the way in which the spectrum of the samples was obtained, the model considers a very high number of samples to be outliers. It should be noted that this parameter has a high number of samples with a zero concentration, which, despite being introduced as a value when developing the model, have been eliminated because they show abnormally high residuals of predicted versus reference values (T criteria). This issue has already been highlighted by Lohr et al. [67] for the calibration of starch and TNC in leaves of ornamental cuttings. For this reason, despite the high RSQ value obtained for fructose, this cannot be considered directly as a good indicator of the quality of the model obtained because the concentration range of the parameter can make the RSQ value misleading, as pointed out by Cozzolino et al. [68]. In addition, the interpretation of the RSQ depends on the distribution of the analytical values. Although normality of the data is a requirement for the calculation of regression models, models with non-normally distributed data can also be developed although they are more difficult to interpret and require more latent variables than models with normally distributed data [69]. 

### 3.4. Validation

The calibration models were validated primarily based on cross-validation. The aim of this validation is to check the predictive ability of the model and to ensure that the model does not overfit the training data, which could lead to poor performance in new samples [70]. For this purpose, the calibration dataset was divided into four groups and each group was used to predict its value from the calibration developed with the other samples. The process was repeated four times so that all groups were used for calibration and prediction. Once the process was completed, the parameters of the multiple correlation coefficient in validation (RSQ_val_) and standard error of prediction (SEP) were considered to select the best model. 

**Figure 2:**
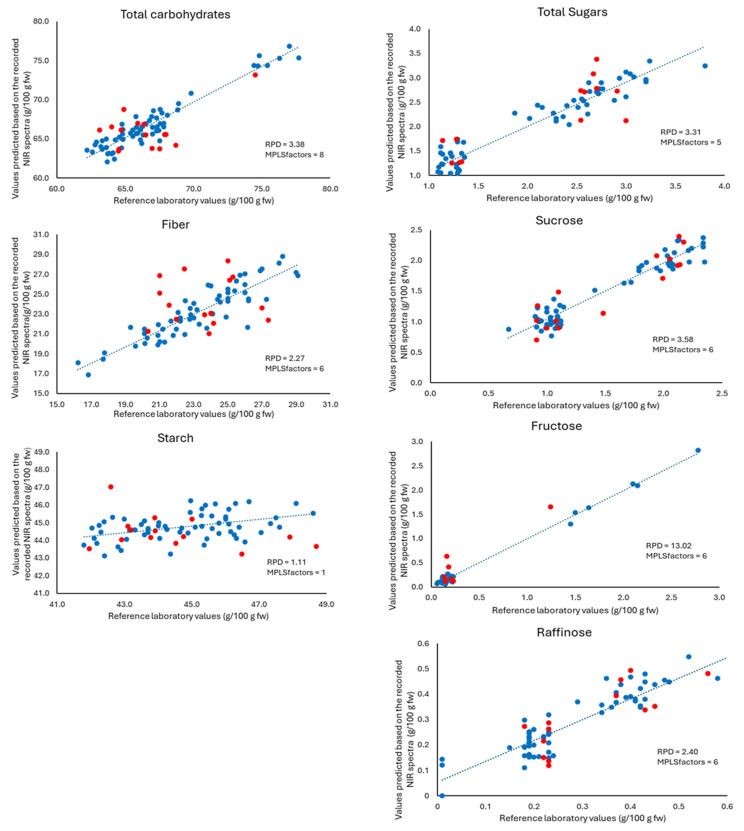
Correlation between the values obtained in the reference methods and the values predicted based on the recorded NIR spectra, for records made on whole lentils. Cross-validation samples (blue) and External validation samples (red). RPD: ratio performance deviation, Modified Partial Least Squares (MPLS): number of factors in the prediction equation.

**Figure 3:**
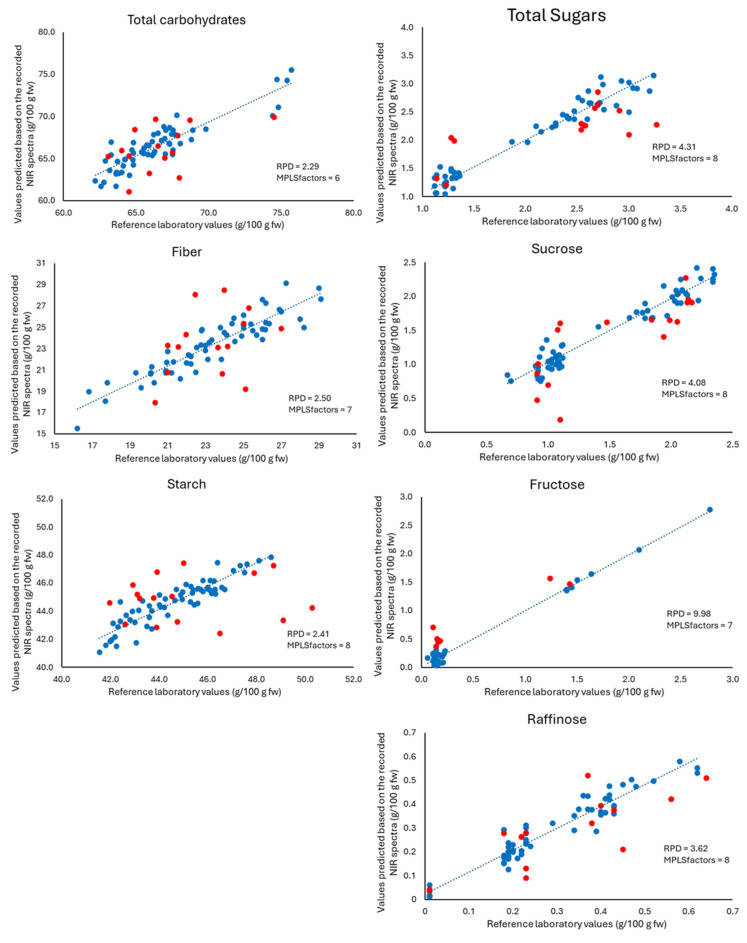
Correlation between the values obtained in the reference methods and the values predicted based on the recorded NIR spectra, for recordings made on ground lentils. Cross-validation samples (blue) and External validation samples (red). RPD: ratio performance deviation, Modified Partial Least Squares (MPLS): number of factors in the prediction equation.

Subsequently, an external validation was carried out. For this purpose, the calibration equations obtained were applied to the spectra of the 16 samples that did not participate in the development of the models. The results obtained in the cross-validation and external validation can be seen in Figure 2 and Figure 3, for whole-recorded lentils and ground-recorded lentils, respectively. 

The RSQ_val_ value informs us of the correlation between the value calculated by the model and the value obtained in the laboratory analysis. The results reveal that, after the cross-validation process, no change in RSQ values is observed in the ground lentils (Table 2). However, in whole lentils, the values for total carbohydrates were improved while in the case of fibre, the RSQ_val_ (0.806) is lower than that obtained in the model. As already indicated, the recording of samples after milling and sieving provides better results than the recording of whole samples except for the parameter total carbohydrates. The SEP obtained for ground samples were lower than those obtained for whole ones. The RPD values were higher than 2.5, which allows us to establish that our models were good for all parameters except total carbohydrates. 

Another aspect to be considered is the number of factors in the generated equation. In our case, the number of factors for the best equation ranged from 1 for starch to 8 for carbohydrates in whole samples. In the case of grounded samples, it ranged from 6 for total carbohydrates and 8 for starch, total sugars, fructose, and sucrose. The selection of the number of factors is important to take advantage of all the information provided by the spectra without introducing noise to the model [62]. However, a large variability of terms has been described in the bibliography. Thus, previous studies have described 20 terms in an equation (RSQ = 0.96) for the determination of crude protein in elephant grass [57], 16 terms for equations (RSQ = 0.78) for the prediction of total solids in watermelon [50], 7 terms for an equation (RSQ = 0.91) for the prediction of cannabinol in Cannabis [62] or 2 terms in an equation (RSQ = 0.81) for the detection of potassium in wine [68]. In relation to carbohydrates, Choung [58] pointed out six terms for an equation (RSQ = 0.94) for sucrose prediction and two terms in a non-useful equation (RSQ < 0.3) for raffinose prediction. In the case of starch, 15 terms were used for an equation (RSQ = 0.7) on leaves of ornamentals [67]. The results obtained for total sugars and individual sugars (sucrose and raffinose) are noteworthy. The low moisture content of the lentils could explain the good results obtained in this study. Previous studies have shown that the heterogeneity and water content of the samples can make the NIR calibration of these sugars not possible [67]. The data obtained in the external validation were also analysed using a Student’s *t*-test for paired values (Table A3). The *p*-values obtained were above the minimum level of significance (0.05). Therefore, it can be stated that there is no difference between the values predicted by the models developed, and the values obtained by the different reference methods indicate that these models are suitable for semi-quantitative to quantitative classification of carbohydrates in lentils. However, the case of fructose must be considered with caution (*p* > 0.05 for ground lentils) due to the high number of samples that the model eliminates as outliers. This resulted in only a small number of samples with high amounts of fructose in both the calibration and validation set (Table 2), therefore a high uncertainty in the NIR prediction must be considered in these cases.

There are wavelengths that have a greater influence on the predictive ability of the developed models [71]. As indicated in the materials and methods section, the beta coefficients of the equations allow us to know what these wavelengths are. The wavelengths and coefficients obtained in this study are shown in the Appendix A (Table A4). The wavelength range between 1616 and 1986 nm offers the highest correlation with the concentration of carbohydrates analysed in lentils, with differences being observed depending on whether the samples are recorded whole or ground. It is noted that there are four wavelength intervals that show a significant correlation with the concentration of the parameters analysed. Thus, the first interval covers the wavelengths between 1616 and 1666 nm. These absorbances are mainly related to the concentration of total sugars when the samples are recorded whole, while they are related to fibre and total carbohydrates when the samples are ground. A second interval is located between 1716 and 1798 nm and correlates with the concentration of starch, fructose, total carbohydrates and raffinose, provided that the samples are recorded whole. Of note is the quantification of starch, fructose and raffinose; all five coefficients influencing their quantification are in this range, which seems to indicate a strong association between this wavelength and these compounds when samples are recorded whole. The third interval comprises the wavelengths between 1800 and 1862 nm, which shows a strong correlation with the concentrations of starch, sucrose, and raffinose if the samples are recorded ground. Finally, a fourth interval is located between 1926 and 1986 nm and correlates with the concentrations of all the parameters analysed in the ground samples except for raffinose and sucrose. Moreover, it is a range with a strong influence on the quantification of fibre and sucrose when samples are recorded whole. 

## 4. Conclusions

All lentil varieties tested showed high carbohydrate contents with starch being the major compound. Among the individual sugars, sucrose is the main sugar, while the presence of other sugars depends on the variety analysed. Statistically significant differences between varieties were found for all compounds. The methodology of determining the carbohydrate composition of lentils by NIR spectroscopy has been studied. The recorded spectra for both whole and ground lentils showed three zones of maximum absorption in the intervals 1200–1250 nm, 1440–1500 nm, and 1900–1936 nm. Internal and external cross-validation of the developed models showed good predictive capability, with adequate RSQ and RPD values for most parameters. Grounding and sieving of the sample prior to NIR recording reduced the scattering of the recorded spectra and improved the predictive ability of the models. Specific wavelengths were identified that showed a high correlation with the concentration of the analysed parameters. These wavelengths varied depending on the parameter and recording method. Due to the structure and distribution of carbohydrates in lentils, the determination of starch was only possible in the ground samples. Also, the low concentrations and absence of fructose in some lentil varieties make a larger data set necessary for the development of a more stable model for this parameter. The results obtained confirm that the information collected in the NIR spectra of lentils allows them to be applied for the quantification of the carbohydrate content in lentils. Near-infrared spectroscopy (NIRS) shows promise as a rapid and non-destructive method to assess carbohydrates in lentils to support breeding and product development.

## Figures and Tables

**Table 1:** Carbohydrate composition and moisture of lentil samples (g/100 gfw) determined by reference methods (mean ± standard deviation).

Lentil Cultivars						
	Macrosperm		Microsperm			
	Guareña	Castellana	Pardina	Crimson	Beluga	Stone
TCH *	66.59 ± 1.54 _a_	75.46 ± 0.79 _b_	64.87 ± 1.63 _a_	76.10 ± 0.70 _b_	74.86 ± 0.72 _b_	76.50 ± 1.50 _b_
Fibre	22.82 ± 2.26 _c_	16.32 ± 1.31 _b_	24.67 ± 2.15 _c_	12.62 ± 1.15 _a_	25.13 ± 2.12 _c_	18.07 ± 1.40 _b_
Starch	44.32 ± 1.58 _ab_	43.03 ± 2.10 _a_	45.14 ± 1.88 _ab_	48.73 ± 2.54 _c_	43.63 ± 2.33 _ab_	46.70 ± 1.40 _bc_
Total Sugars	2.61 ± 0.28 _c_	2.22 ± 0.16 _b_	1.23 ± 0.08 _a_	3.22 ± 0.11 _d_	3.26 ± 0.57 _d_	3.40 ± 0.35 _d_
Fructose	0.15 ± 0.04 _a_	1.48 ± 0.03 _b_	nd	1.42 ± 0.02 _b_	1.43 ± 0.20 _b_	2.34 ± 0.38 _c_
Sucrose	2.04 ± 0.20 _b_	0.76 ± 0.13 _a_	1.02 ± 0.08 _a_	1.83 ± 0.06 _b_	1.84 ± 0.36 _b_	1.05 ± 0.05 _a_
Raffinose	0.43 ± 0.09 _c_	0.01 ± 0.00 _a_	0.21 ± 0.02 _b_	0.01 ± 0.00 _a_	nd	nd
Moisture	7.12 ± 0.93 _a_	8.74 ± 0.59 _ab_	7.86 ± 1.33 _ab_	8.98 ± 0.27 _ab_	9.39 ± 0.45 _b_	9.23 ± 0.10 _ab_

* TCH: Total carbohydrates, nd: no detected; _a–d_: values followed by different subscripts in the same row are significantly different (*p* < 0.05).

**Table 2:** Statistical descriptors for the best Modified Partial Least Squares calibration equations obtained from NIR spectra (1100–2000 nm) and carbohydrate composition on lentil samples.

	Math Treatment	n	T	RSQ	SEC	SECV	RPD	RSQ_val_	SEP	RSQ_p_
Whole lentil										
TC	None 2, 4, 4, 1	61	8	0.898	1.173	2.847	3.144	0.912	1.083	0.285
Fiber	SNV-D 2, 4, 4, 1	59	4	0.828	1.222	2.640	2.414	0.806	1.288	0.540
Starch	SNV 1, 10, 10, 1	60	5	0.174	1.543	1.595	1.100	0.188	1.517	0.303
Total Sugars	SNV-D 2, 4, 4, 1	60	8	0.900	0.247	0.460	3.163	0.909	0.234	0.676
Fructose	SNV 2, 4, 4, 1	33	8	0.993	0.061	0.257	11.834	0.994	0.055	0.943
Sucrose	None 2, 4, 4, 1	59	7	0.913	0.158	0.278	3.389	0.922	0.149	0.832
Raffinose	SNV-D 2, 4, 4, 1	61	6	0.847	0.060	0.103	2.562	0.829	0.058	0.657
Ground lentil										
TC	Detrend 2, 4, 4, 1	58	8	0.787	1.465	3.064	2.167	0.809	1.375	0.340
Fiber	SNV 2, 4, 4, 1	60	6	0.819	1.250	2.720	2.351	0.840	1.164	0.250
Starch	SNV 2, 4, 4, 1	61	7	0.802	0.786	1.964	2.248	0.828	0.726	0.300
Total Sugars	None 2, 4, 4, 1	57	7	0.937	0.185	0.398	3.997	0.946	0.169	0.586
Fructose	None 2, 4, 4, 1	34	10	0.984	0.087	0.423	7.926	0.990	0.071	0.920
Sucrose	None 2, 4, 4, 1	60	12	0.930	0.142	0.335	3.802	0.940	0.131	0.629
Raffinose	None 2, 4, 4, 1	60	8	0.917	0.044	0.109	3.482	0.925	0.041	0.306

TC: Total Carbohydrates, n: number of samples after removing the outliers; T: PLS terms; SEC: Standard Error of Calibration; RSQ: Multiple Correlation Coefficient; RPD: Ratio Performance Deviation; SNV: Standard Normal Variate only; SNV-D: Standard Normal Variate combined with Detrend, RSQ_val_: multiple correlation coefficient in cross-validation, SEP: standard error of prediction, RSQ_p_: multiple correlation coefficient in prediction.

## Data Availability

The data presented in this study are available on request from the corresponding author.

## Appendix A

**Table A1:** Lentil samples analysed in this study with indication of cultivar.

Sub-Specie	Cultivar	Sample Number
Macrosperm	Guareña	37
	Castellana	
Microsperm	Pardina	43
	Crimson	
	Beluga	
	Stone	

**Table A2:** Main carbohydrate composition (g/100 gfw) of the samples of lentil in the set of calibration and validation.

	Calibration Set (n = 64)			External Validation Set (n = 16)		
	Min-Max	Mean	SD	Min-Max	Mean	SD
TCH *	62.20–77.70	67.27	4.12	63.10–75.30	67.08	3.45
Fiber	11.62–29.11	22.89	3.55	12.37–27.36	22.89	3.50
Starch	41.54–50.10	44.78	1.86	41.96–50.30	45.13	2.57
Total Sugars	1.09–3.84	2.09	0.82	1.13–3.27	2.09	0.81
Fructose	0.00–2.78	0.33	0.63	0.00–1.43	0.24	0.43
Sucrose	0.67–2.35	1.49	0.54	0.91–2.17	1.56	0.53
Raffinose	0.01–0.62	0.26	0.16	0.01–0.64	0.30	0.17

* TCH: Total carbohydrates.

**Table A3:** External validation of the equations shown in Table 2 for each of the recording methods and parameters analyzed. Root mean standard error (RMSE) and comparison statistic between the values obtained in the samples by the reference methods and those predicted by the developed model.

	Whole Lentil		Ground Lentil	
Parameter	RMSE	t-Student (*p* < 0.05)	RMSE	t-Student (*p* < 0.05)
TCH *	2.513	0.432	4.715	0.126
Fiber	4.808	0.433	3.648	0.736
Starch	3.619	0.664	2.877	0.711
Total Sugars	0.731	0.889	0.574	0.137
Fructose	0.575	0.207	0.339	0.030
Sucrose	0.226	0.793	0.384	0.100
Raffinose	0.086	0.327	0.155	0.790

* TCH: Total carbohydrates.

**Table A4:** Wavelengths (λ) in nm associated with the five coefficients (*β*) with the greatest weight in the quantification obtained from the equations listed in Table 2 for each of the parameters analysed in whole and ground lentils.

Fiber		Starch		Total Carbohydrates		Total Sugars		Fructose		Sucrose		Raffinose	
Whole lentils													
λ	*β*	λ	*β*	λ	*β*	λ	*β*	λ	*β*	λ	*β*	λ	*β*
1976	139,760.2	1730	16.9	1780	7,446,145.8	1482	5970.1	1534	620.4	1824	32,047.2	1676	4082.8
1508	95,451.8	1728	19.1	1816	6,763,281.0	1484	4924.5	1514	599.9	1822	29,554.5	1718	4064.2
1978	91,068.6	1762	15.4	1626	6,278,431.7	1480	4857.1	1718	586.7	1832	27,229.3	1500	3930.2
1974	87,621.2	1760	15.3	1516	5,391,850.7	1478	3313.4	1536	583.2	1974	22,553.1	1678	3904.6
1510	63,117.5	1732	14.3	1782	4,961,843.1	1486	2491.2	1716	579.5	1540	22,427.5	1824	3548.6
Ground lentils													
λ	*β*	λ	*β*	λ	*β*	λ	*β*	λ	*β*	λ	*β*	λ	*β*
1496	235,719.2	1822	102,070.2	1564	9,884,031.1	1956	226,788.1	1506	232,391.3	1554	17,395.1	1554	17,395.1
1498	177,436.6	1496	745,777.0	1958	9,793,927.3	1958	206,738.6	1508	150,347.3	1556	11,292.8	1556	11,292.8
1494	126,406.8	1530	70,060.5	1956	8,947,196.6	1954	202,936.3	1504	145,112.3	1546	11,021.4	1546	11,021.4
1526	104,437.3	1824	67,829.8	1562	7,924,359.5	1582	194,105.1	1962	103,510.1	1802	9455.3	1802	9455.3
1630	102,843.8	1494	63,871.5	1798	7,708,290.9	1806	179,468.9	1960	98,024.0	1552	9272.6	1552	9272.6
